# Novel approach of prophylactic radiation to reduce toxicities comparing 2-step40 with 56-Gy simultaneous integrated boost intensity-modulated radiation therapy for locally advanced squamous cell carcinoma of the head and neck, an intergroup phase III trial (JCOG1912, NEW BRIDGE)

**DOI:** 10.1186/s12885-023-11503-z

**Published:** 2023-11-06

**Authors:** Tomoya Yokota, Sadamoto Zenda, Takeshi Kodaira, Naomi Kiyota, Yasushi Fujimoto, Koichiro Wasano, Ryo Takahashi, Takashi Mizowaki, Akihiro Homma, Keita Sasaki, Ryunosuke Machida, Yuta Sekino, Haruhiko Fukuda, Japan Clinical Oncology Group Radiation Therapy Study Group, Japan Clinical Oncology Group Head and Neck Cancer Study Group

**Affiliations:** 1 Division of Gastrointestinal Oncology, Shizuoka Cancer Center, Sunto-gun, Japan; 2 Department of Radiation Oncology, National Cancer Center Hospital East, Kashiwa, Japan; 3 Department of Radiation Oncology, Aichi Cancer Center Hospital, Nagoya, Japan, 1-1 Kanoko-den, Chikusa-ku, Nagoya, Aichi 464-8681 Japan; 4 Department of Medical Oncology and Hematology, Cancer Center, Kobe University Hospital, Kobe, Japan; 5 Department of Otolaryngology, Aichi Medical University, Nagakute, Japan; 6 Department of Otolaryngology-Head and Neck Surgery, Tokai University School of Medicine, Isehara, Japan; 7 Section of Radiation Safety and Quality Assurance, National Cancer Center Hospital East, Kashiwa, Japan; 8 Departments of Radiation Oncology and Image-Applied Therapy, Kyoto University Graduate School of Medicine, Kyoto, Japan; 9 Department of Otolaryngology-Head and Neck Surgery, Faculty of Medicine, Graduate School of Medicine, Hokkaido University, Sapporo, Japan; 10 Japan Clinical Oncology Group Data Center/Operations Office, National Cancer Center Hospital, Tokyo, Japan

**Keywords:** Locally advanced head and neck cancer, Chemoradiotherapy, Intensity modulated radiation therapy, Prophylactic radiation, 2-step, Simultaneous integrated boost, Randomized controlled study

## Abstract

**Background:**

Chemoradiotherapy (CRT) with concurrent cisplatin is the standard of care as a nonsurgical definitive treatment for patients with locally advanced squamous cell carcinoma of the head and neck (LA-SCCHN). However, CRT is associated with increased severe late adverse events, including swallowing dysfunction, xerostomia, ototoxicity, and hypothyroidism. Few strategies aimed at less invasive CRT without compromising treatment outcomes have been successful. The purpose of this study is to confirm the non-inferiority of reduced dose prophylactic radiation with 40 Gy compared to standard dose prophylactic radiation with 56 Gy in terms of the time to treatment failure (TTF) among patients with clinical stage III-IVB LA-SCCHN.

**Methods:**

This study is a multicenter, two-arm, open-label, randomized phase III trial. Patients with LA-SCCHN excluding p16 positive oropharynx cancer are randomized to the standard arm or experimental arm. A total dose of 70 Gy for tumors with concurrent cisplatin at 100 mg/m^2^ are administered in both arms. For prophylactic field, patients in the standard arm receive a total dose of 56 Gy in 35 fractions for 7 weeks using simultaneous integrated boost (SIB56) and those in the experimental arm receive 40 Gy in 20 fractions using two-step methods for 4 weeks (2-step40). A total of 400 patients will be enrolled from 52 Japanese institutions within 5 years. The primary endpoint is TTF, and the secondary endpoints are overall survival, complete response rate, progression-free survival, locoregional relapse-free survival, acute and late adverse events, quality of life score, and swallowing function score.

**Discussion:**

If the experimental arm is non-inferior to the standard arm in terms of TTF and superior on the safety endpoints, the 2-step40 procedure is the more useful treatment than SIB56 for definitive CRT.

**Trial registration:**

This trial has been registered in the Japan Registry of Clinical Trials as jRCTs031210100 (https://jrct.niph.go.jp/latest-detail/jRCTs031210100). Date of Registration: May 2021.

**Supplementary Information:**

The online version contains supplementary material available at 10.1186/s12885-023-11503-z.

## Background

Chemoradiotherapy (CRT) with concurrent high-dose cisplatin (CDDP) is a standard treatment for patients with locally advanced squamous cell carcinoma of the head and neck (LA-SCCHN) [1–4]. However, CRT is associated with increased severe late adverse events, including swallowing dysfunction, nephrotoxicity, neurotoxicity, ototoxicity, and hypothyroidism [5–7], most of which are irreversible and likely to reduce quality of life in patients treated with CRT [8]. Furthermore, long-term results of the Intergroup Radiation Therapy Oncology Group 91 − 11 study revealed an unexplained increase in deaths unrelated to cancer in patients who received concomitant cisplatin plus radiotherapy (RT) [9].

Intensity-modulated radiation therapy (IMRT) has emerged as a current standard method for definitive CRT for LA-SCCHN. Although the advantage of IMRT is its highly conformal dose distribution to the primary tumor and involved lymph nodes with sufficiently low exposure to organs at risk, severe late adverse events after CRT remain a significant problem to be resolved.

Several strategies aiming at less invasive CRT using IMRT for LA-SCCHN have been investigated, including (i) reducing the total irradiation dose, (ii) alternative concurrent pharmacotherapy to CDDP, and (iii) reducing the irradiation dose in prophylactic field. However, an increase in local recurrence by using reduced total dose radiation was noted even in HPV-positive oropharyngeal cancer with favorable clinical outcomes [10, 11]. The combination of cetuximab, an epidermal growth factor receptor − targeting monoclonal antibody, and radiation therapy had inferior therapeutic efficacy to CDDP plus RT, with the similar frequency of feeding tube dependency to CRT because of the high incidence of mucositis [12, 13]. Therefore, we focus on dose reduction in prophylactic field irradiation.

A randomized controlled trial was conducted to investigate reduction of the dose of RT to the elective nodal sites, with 50 Gy in the standard arm and 40 Gy in the experimental arm. The results revealed a trend toward less dysphagia at 6 months in the 40 Gy arm though not statistically significant (38.4% vs. 48.6%), without compromising outcome and survival [14]. However, a large heterogeneity remains in terms of irradiation schedule and prescription, and the use of concurrent chemotherapy.

Based on these backgrounds, we have launched a randomized controlled trial to confirm the non-inferiority of reduced dose prophylactic radiation with 40 Gy using two-step40 IMRT method (2-step40) to standard dose prophylactic radiation with 56 Gy using simultaneous integrated boost (SIB56) in terms of the time to treatment failure for patients with clinical stage III-IVB (except N3a) LA-SCCHN according to UICC-TNM 8th .

## Methods/design

### Study design

This study is a multi-institutional, two-arm, open-label, randomized phase III study. The study protocol was reviewed by the Japan Clinical Oncology Group (JCOG) Protocol Review Committee and approved by as the JCOG1912 (NEW BRIDGE) in January 2021. The certificated review board approved in March 2021, and patient accrual was started at May 2021. The trial registry number is jRCTs031210100 (https://jrct.niph.go.jp/latest-detail/jRCTs031210100).

### Endpoints

The primary endpoint is time to treatment failure (TTF) 1 in all randomized patients. TTF1 is defined as time from randomization to the date of progression, or determination of residual disease after the completion of the protocol treatment, or death from any cause, whichever occurred first, and it is censored at the last day the patient is alive without any evidence of progression. The secondary endpoints are overall survival (OS), complete response rate, progression-free survival (PFS), locoregional relapse-free survival, TTF2, acute and late adverse events, quality of life (QOL) score, and swallowing function score. OS is defined as the time from randomization to death from any cause and is censored on the last contact day for a surviving patient. PFS is defined as the time from randomization to the earliest date of progression or death from any cause and is censored on the last contact day when the patient is alive without any evidence of relapse. In the definition of TTF2, clinical residual disease in TTF1 events is not regarded as an event or censor of TTF2, if complete resection is conducted by salvage surgery and subsequent histopathological examination reveals pathological complete response.

### Eligibility criteria

The inclusion criteria are as follows:

(i) Primary lesions are located in the oropharynx, hypopharynx, or larynx.
(ii) Histologically proven squamous cell carcinoma on biopsy specimens of the primary lesion. Immunohistochemistry reveals p16 negativity in patients with oropharyngeal cancer.
(iii) Clinical stage of III, IVA, or IVB (excluding N3a) based on the 8th UICC-TNM classification.
(iv) Age of ≥ 20 years.
(v) Eastern Cooperative Oncology Group performance status of 0 or 1.
(vi) Measurable lesions are not required.
(vii) No prior chemotherapy for any cancers and no prior RT for brain, head or neck lesions. No prior surgery for head and neck cancers, except for a biopsy of neck lymph nodes for a diagnosis.
(viii) Any of the following conditions:

- Both the primary lesion and regional lymph node metastases are technically or functionally unresectable. Tumor resectability will be determined through a review by a head and neck surgeon.
- Although the tumors are resectable, patients do not wish to undergo surgical resection.

(ix) Adequate organ function.

- Neutrophil ≥ 1,000/mm^3^.
- Hemoglobin ≥ 9.0 g/dL.
- Platelet ≥ 100,000/mm^3^.
- Total bilirubin ≤ 2.0 mg/dL.
- Aspartate aminotransferase ≤ 100 U/L.
- Alanine aminotransferase ≤ 100 U/L.
- CCr ≥ 60 mL/min.

(xxiv) Written informed consent for participation.

Exclusion criteria.

(i) Synchronous or metachronous (within 5 years) double cancers, except for intramucosal tumors curatively resected with local therapy.
(ii) Active infection requiring systemic therapy.
(iii) Fever over 38.0 °C.
(iv) Female during pregnancy, within 28 days of postparturition, or during lactation. Male who wants partner’s pregnancy.
(v) Psychiatric disorder difficult to participate in this clinical study.
(vi) Requirement of systemic steroid medication or immunosuppressant treatment.
(vii) Poorly controlled diabetes mellitus.
(viii) Poorly controlled hypertension.
(ix) Unstable angina within 3 weeks or with a history of myocardial infarction within 6 months before trial enrollment.
(x) Poorly controlled valvular disease, dilated cardiomyopathy, or hypertrophic cardiomyopathy.
(xi) Positive hepatitis B surface antigen test.
(xii) Positive human immunodeficiency virus antibody test.
(xiii) Inability to quit smoking and drinking.

### Randomization

After eligibility has been confirmed, registration is performed using a web-based system at the JCOG Data Center. Patients are randomized to the standard arm (SIB56) or experimental arm (2-step40). Randomization is stratified according to the institution, presence or absence of lymph nodes metastasis, and the primary sites (oropharynx vs. hypopharynx vs. larynx).

### Treatment methods

**Fig. 1:**
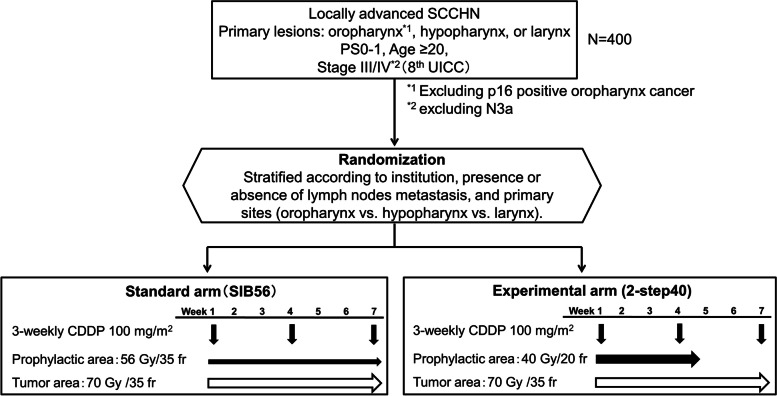
Study scheme. SCCHN, squamous cell carcinoma of the head and neck; CDDP, cisplatin; SIB, simultaneous integrated boost

Patients in both arms receive definitive CRT with CDDP at a dose of 100 mg/m^2^ on days 1, 22, and 43. IMRT is administered at a total dose of 70 Gy given for tumor in single, daily, 2 Gy fractions for 7 weeks in both arms. For prophylactic field, patients in the standard arm receive a total dose of 56 Gy in 35 fractions for 7 weeks, and those in the experimental arm receive 40 Gy in 20 fractions for 4 weeks (Fig. 1) [15].

Radiation planning is performed 2 weeks before the start of RT. The cervical computed tomography (CT) is performed using a thermoplastic mask for fixation. In the 2-step40 method group, another planning CT scan is performed at a dose of 30 Gy per the boost IMRT plan. RT is delivered with 4–10 MV photons using IMRT at a total dose of 70 Gy in 35 fractions over 7 weeks in both arms. The gross tumor volume (GTV) is defined as the volume of the primary tumor and the metastatic lymph nodes measuring 10 mm along the short axis. The clinical target volume (CTV) includes the GTV with a 0.5- 1- cm margin. The regional lymph nodes include the bilaterally cervical lymph nodes, and details on the prophylactic field are determined according to the primary site [16]. The planning target volume (PTV) is defined as CTV plus a 0.5-cm margin to account for organ motion and daily set-up errors. Organs at risk (OAR) was defined as the spinal cord, brainstem, parotid gland, mandible, inner ear, thyroid gland, oral cavity, pharyngeal constrictor muscle, and larynx. The dose constraint of PTV and OAR are defined in the protocol, and quality assurance of radiation planning of all registered patients is mandatory.

### Assessment and follow-up

Before CRT, routine oral screening by dentists, head and neck endoscopy, and esophagogastroduodenoscopy are performed. All patients should receive baseline evaluation of swallowing function, hearing function, and QOL. Swallowing function is assessed by performing a videoendoscopic evaluation of swallowing and using Functional Outcome Swallowing Scale (FOSS). The hearing function is evaluated using pure-tone audiometry. The Core Quality of Life questionnaire (QLQ-C30) and Quality of Life Questionnaire Head and Neck Module (QLQ-HN43) scales are used for the QOL evaluation.

During protocol treatment, evaluations of general and performance statuses, safety profile according to the Common Terminology Criteria of Adverse Events (CTCAE) version 5.0, and blood examinations are performed at least weekly.

The initial efficacy assessment using enhanced CT for the cervix, chest, and abdomen, and magnetic resonance imaging (MRI) of the head and neck region are performed 16 weeks (± 4 weeks allowed) after the start of treatment. If there is a contrast effect secondary to tumor shrinkage (scarring), Positron Emission Tomography (PET)-CT should be performed 4 weeks (± 1 week allowed) after the initial efficacy evaluation because of the difficulty in determining efficacy. A biopsy is recommended if macroscopic residual disease is suspected. If the biopsy is positive, or if there is positive FDG uptake by PET-CT as well as corresponding lesions confirmed on MRI/CT, or if there is evidence of progression, salvage surgery is considered. Primary lesion excision and/or neck dissection is performed as salvage surgery depending on the lesion.

Patients’ general status, acute and late toxicities, blood examination including thyroid function, enhanced CT and MRI are periodically performed every 16 weeks in the first 2 years, and every 24 weeks from the second to the third year after the initiation of protocol treatment. Evaluations of swallowing and QOL are performed at 16 weeks, and 1, 2, and 3 years after the initiation of the protocol treatment. Evaluation for hearing function is performed at 1 year and 3 years after initiation of protocol treatment. The precise assessment schedule from pretreatment to 3 years after initiation of the protocol treatment is shown in Table 1.

**Table 1:** Study calendar

	Pretreatment	During treatment	− 90D^b^	91D − 2Y^c^	2Y – 3Y^d^	16 W	1Y	2Y	3Y
Physical examination	○	△^1 W^	◎	△^16 W^	△^24 W^				
PS	○	△^1 W^	◎	△^16 W^	△^24 W^				
CT/MR	○			△^16 W^	△^24 W^				
Blood examination^a^	○	△^1 W^	◎	△^16 W^	△^24 W^				
Safety profile		△^1 W^	◎	△^16 W^	△^24 W^				
Body weight	○	○	◎			○	○	○	○
Nutrition access	○						○	○	○
Swallowing function	○					○	○	○	○
Pure Tone Audiometry	○						○		○
QOL	○					○	○	○	○

^a^Including thyroid stimulating hormone (TSH) and thyroxine

^b^From end of protocol treatment to 90 days after initiation of protocol treatment

^c^From 91 days to 2 years after initiation of protocol treatment

^d^From 2 years to 3 years after initiation of protocol treatment

*CT *Computed tomography, *MRI *Magnetic resonance imaging, *QOL *Quality of life

△1 W: every week, △16 W: every 16 week,△24 W:every 24 week, ◎;at least every 2 week within 28 days after the end of chemoradiotherapy, but at least every 4 week 29 days after the end of chemoradiotherapy

### Statistical analysis and evaluation criteria for the endpoints

A 3-year TTF1 of 50% is assumed for both arms. According to the Schoenfeld and Richter’s method [17], the required sample size has been calculated as 197 patients per arm, with a study-wise one-sided alpha level of 5%, a power of 75%, and a non-inferiority margin of 10% (hazard ratio = 1.32). A total of 400 patients will be included within 5 years, and all randomized patients will be followed up for at least 5 years after the patient recruitment is completed. The primary endpoint is analyzed 3 years after recruitment has been completed. All statistical analyses will be conducted at the JCOG Data Center.

If the experimental arm is non-inferior to the standard arm in terms of TTF1 and superior in terms of safety endpoints, the 2-step40 method will be considered the more efficacious treatment. However, it is unacceptable that a point estimate of 3-year OS in the experimental arm is more than 5% lower than that in the standard arm. Based on mathematical models, the rates of key late adverse events of grade 2 or higher are expected to be reduced by approximately 15%, 7%, 7%, and 10% for hearing impairment, hypothyroidism, dry mouth, and dysphagia, respectively. If the experimental arm shows sufficient improvement in at least one of these key late adverse events, the study treatment will be considered as beneficial. If the non-inferiority of the experimental arm in terms of the primary endpoint is not demonstrated, the SIB56 method will be considered a useful treatment.

### Interim analysis and monitoring

Two interim analyses considering multiplicity will be conducted using the Lan and DeMets method along with the O’Brien and Fleming-type alpha spending function [18]. The first interim analysis will be conducted after half of the planned number of patients are enrolled, and the second interim analysis will be performed after the planned patient recruitment is completed. The Data and Safety Monitoring Committee of the JCOG will review the interim analysis report independently from the group investigators and group statisticians.

In the first interim analysis, if both the non-inferiority and superiority of the experimental arm over the standard arm in terms of the primary endpoint are demonstrated with an adjusted alpha level, the study will be terminated. If the non-inferiority of the experimental arm at the primary endpoint is not demonstrated or its non-inferiority but not superiority is demonstrated, the study will be continued. In the second interim analysis, the study will be terminated if the non-inferiority of the experimental arm to the standard arm in the primary endpoint is demonstrated with an adjusted alpha level. If the non-inferiority of the experimental arm in the primary endpoint is not demonstrated, the study will be continued. In both interim analyses, if the point estimate of the hazard ratio of TTF1 exceeds the non-inferiority margin (1.32), the study will be terminated because of futility.

In-house monitoring will be performed every 6 months by the JCOG Data Center to evaluate and improve study progress, data integrity, and patient safety. All enrolled patients are evaluated for compliance with the RT protocol at the time of completion of RT. The purpose of this assessment is to confirm whether the actual treatment at each participating institution is performed according to the protocol regulations and to provide feedback for the treatment of subsequently enrolled patients.

### Cases review

Cases of patients who undergo salvage surgery after the completion of the protocol treatment will be presented at the JCOG Head and Neck Cancer Study Group conference held every 6 months to review indications for surgery and the appropriateness of the surgical procedure. Cases with regional recurrence will be reviewed at the JCOG Radiation Therapy Study Group conference every 6 months to investigate whether the regional recurrences occur inside or outside the elective nodal sites. The RT plan, imaging data, and clinical course during the protocol treatment will be evaluated in each case.

## Discussion

The current CRT regimen for patients with LA-SCCHN is associated with severe late adverse events, decreased QOL, and increased noncancer-related deaths in long-term survivors. The ideal treatment for patients with LA-SCCHN is a modality that is satisfactory for both disease control and the patients’ daily lives. Our trial addresses the question of whether a reduced dose of RT to the prophylactic field without reducing the dose to the target lesion and a concurrent CDDP dose is non-inferior to standard-dose prophylactic RT. Reducing the dose to the prophylactic field is expected to reduce late toxicity by minimizing the irradiation of normal organs.

If our study demonstrates that the experimental arm could achieve fewer adverse events without a reduction in efficacy, reduced-dose prophylactic IMRT could be a new standard method of definitive CRT for LA-SCCHN. Numerous clinical studies have investigated the combination strategy of RT and immune checkpoint inhibitors (ICIs). However, the JAVELIN head and neck 100 trial [19] and KEYNOTE-412 [20] study failed to show the superiority of programmed death-ligand 1 (PD-L1) or programmed cell death protein 1 (PD-1) inhibitors combined with CRT over CRT alone. Recent preclinical research suggests a role for the draining lymph nodes (DLNs), where T-cell priming and activation occur, in antitumor immunity [21–25]. These studies demonstrated that elective nodal irradiation against DLNs resulted in extratumoural immunosuppression through lymph node dysfunction and lymphopenia when the lymph nodes and/or large volumes of circulating blood were within the irradiation field. Therefore, elective lymph node sparing in the experimental arm may be associated with the recovery of synergistic antitumor responses to ICIs. Based on our trial, clinically optimized elective nodal irradiation may enable the development of a combination strategy involving RT and ICIs. However, if the efficacy and minimally invasive nature of the 2-step40 method are not demonstrated, the conventional standard treatment SIB56 treatment method remains the global standard. Thus, both the positive and negative results of this randomized trial will have an impact on the clinical practice. Furthermore, these results can be universally applied as a standard prophylactic irradiation method, even if any regimen involving a novel pharmacotherapy in combination with CRT or RT is developed in a definitive setting.

One significance of this trial is the prospective follow-up for late toxicities, focusing on swallowing function, hearing function, and thyroid functions. Of these, ototoxicity is caused by cochlear toxicity due to CDDP and irradiation, and is irreversible, and is likely to reduce the QOL in patients treated with CRT [6]. Although the American Speech-Language-Hearing Association and the American Academy of Audiology recommend audiological assessments before, during, and after ototoxic drug administration [26, 27], monitoring for ototoxicity in clinical practice remains underutilized in the clinical practice. One study suggested that twice as many people have clinically meaningful changes in baseline hearing on audiograms as those who reported hearing changes [28]. Therefore, the assessment of hearing loss by CTCAE alone, without using a Monitoring Program, may underestimate a large population at risk for long-term effects. Precise ototoxicity monitoring in the protocol of this trial may detect the true incidence, timing, and extent of hearing loss, and clarify whether a cochlear injury occurs before patients subjectively complain of hearing loss. Furthermore, it would be of great significance if de-escalated prophylactic radiation in the experimental arm contributes to an improvement in hearing function.

### Participating institutions

This study is a within-JCOG intersubgroup study collaborating between the Radiation Therapy Study Group and the Head and Neck Cancer Study Group. The participating institutions are those that meet the criteria for RT quality control and quality assurance and have approval from the JCOG Radiation Therapy Committee to perform IMRT. All participants will be recruited from the following 52 hospitals: Saitama Cancer Center, Sapporo Medical University, Aichi Cancer Center, Kindai University Hospital, Hiroshima University, Tochigi Cancer Center, Hyogo Cancer Center, Niigata Cancer Center, Tohoku University, Kobe University, Okinawa Prefectural Chubu Hospital, Yokohama City University Hospital, Osaka International Cancer Institute, Iwate Medical University, National Hospital Organization Tokyo Medical Center, National Canter Center Hospital, Tokai University, Kyoto University, National Hospital Organization Shikoku Cancer Center, Miyagi Cancer Center, Chiba University, Tokyo Medical and Dental University, National Cancer Center Hospital East, Hokkaido University, Tokyo Medical University Hospital, Nara Medical University, Cancer Institute Hospital of the Japanese Foundation for Cancer Research, University of Tokyo, Jichi Medical University, Fujita Health University, Nagoya University, Kyushu University, Saitama Medical University International Medical Center, Showa University, NHO Kyushu Cancer Center, Institute of Medicine, University of Tsukuba, Kansai Medical University Hospital, Tokyo Metropolitan Cancer and Infectious Diseases Center Komagome Hospital, The Jikei University Hospital, Shizuoka Cancer Center, Kitasato University School of Medicine, Okayama University Hospital, Osaka University, Kochi Medical School Hospital, Osaka Metropolitan University Hospital, Juntendo Hospital, Nihon University Itabashi Hospital, Chiba Cancer Center, University of Yamanashi Hospital, Keio University, Shiga General Hospital, and Kanagawa Cancer Center.

### Supplementary Information

**Additional file 1.**

## Data Availability

Not applicable.

## Abbreviations

CRT: Chemoradiotherapy
LA-SCCHN: Locally advanced squamous cell carcinoma of the head and neck
SIB: Simultaneous integrated boost
2-step40: 40 Gy in 20 fractions for 4 weeks in the prophylactic area
TTF: Time to treatment failure
CDDP: Cisplatin
RT: Radiotherapy
IMRT: Intensity-modulated radiation therapy
JCOG: Japan Clinical Oncology Group
QOL: Quality of life
CT: Computed tomography
QLQ-C30: Core Quality of Life questionnaire
QLQ-HN43: Quality of Life Questionnaire Head and Neck Module
ICI: Immune checkpoint inhibitor
PD-1: Programmed cell death protein 1
PD-L1: Programmed death-ligand 1
